# Cannabidiol Supplements in Romania: Bridging the Gap Between Marketed Claims and Clinical Reality

**DOI:** 10.3390/pharmacy12060176

**Published:** 2024-11-25

**Authors:** Corina Andrei, George Mihai Nitulescu, Georgiana Nitulescu, Anca Zanfirescu

**Affiliations:** Faculty of Pharmacy, “Carol Davila” University of Medicine and Pharmacy, Traian Vuia 6, 020956 Bucharest, Romania; corina.andrei@umfcd.ro (C.A.); georgiana.nitulescu@umfcd.ro (G.N.); anca.zanfirescu@umfcd.ro (A.Z.)

**Keywords:** cannabidiol, supplements, online pharmacy, clinical evidence, phytocannabinoid

## Abstract

In recent years, the European market, including Romania, has witnessed a significant increase in the promotion of cannabidiol (CBD)-based products, often presented as effective treatments for various health conditions. This study investigates the inconsistencies between the health claims associated with these supplements and the evidence from clinical trials. To identify products available on the Romanian market, a systematic review of online pharmacies and websites that specialize in selling CBD-based products has been performed. Additionally, a systematic review of clinical trials has been conducted to assess the efficacy of CBD for the specified indications. Our analysis revealed that some claims, such as those related to post-traumatic stress disorder, lack substantial clinical evidence. Moreover, even when clinical support exists, the dosages recommended for the supplements are often significantly lower than those used in trials, raising concerns about their efficacy. These findings highlight the need for stricter regulatory oversight and more transparent communication to ensure that consumer expectations are aligned with scientific evidence, ultimately promoting informed decision-making and consumer safety.

## 1. Introduction

Cannabidiol (CBD) has gained widespread attention for its potential therapeutic effects in treating anxiety, pain, and inflammation [1]. Preclinical research has demonstrated its anxiolytic and antidepressant properties, enhancing fear extinction, reducing anxiety responses, and modulating serotonin and noradrenaline levels [2]. Animal studies indicate that CBD may promote neurogenesis and increase brain-derived neurotrophic factor signaling in the prefrontal cortex, potentially improving mood and cognitive functions [3]. These findings support CBD’s potential as a treatment for anxiety and mood disorders, although more clinical research is necessary to confirm its efficacy. Further investigations into CBD’s peripheral effects show its anti-inflammatory actions, primarily mediated through its indirect interaction with CB_2_ cannabinoid receptors, modulating immune responses, reducing pro-inflammatory cytokine production, and enhancing anti-inflammatory mediators [4]. It also affects various ion channels and receptors like transient receptor potential vanilloid 1 (TRPV1) [5] and peroxisome proliferator-activated receptors (PPARγ) [6], which are crucial in pain modulation [7] and neuroprotection [8,9]. These studies suggest that CBD may alleviate conditions associated with inflammation, chronic pain, and neurodegenerative disorders, highlighting a potential broad therapeutic use. Moreover, the therapeutic potential of CBD has also been evaluated in other pathologies such as autism [10], sleep disorders [11], psychosis [12], substance addiction [13], cancer [14], and low immunity [15]. However, the broad therapeutic applications of CBD remain speculative as these findings have yet to be consistently replicated in clinical settings. As interest in CBD grows, so does the market for supplements, including in Romania. CBD products, from oils for oral administration to suppositories, are now widely sold, often marketed with claims of reducing anxiety, alleviating pain, and improving sleep. This has led to a surge in consumer interest, with many people turning to CBD as a natural remedy [16]. However, despite these bold claims, the previously mentioned significant gap remains between the marketed benefits and the clinical evidence. While global research is promising, the therapeutic effects of CBD remain unproven for many conditions, and specific clinical studies that validate the safety and effectiveness of these products are lacking [16,17].

The therapeutic benefits, efficacy, and safety of CBD have been confirmed for two products: Epidiolex and Sativex. Epidiolex containing 100 mg/mL of CBD, is FDA- and EMA-approved for treating seizures associated with Dravet and Lennox-Gastaut syndromes [18,19]. Sativex (nabiximols) contains both CBD and tetrahydrocannabinol (THC) and is formulated as an oromucosal spray. It is recommended for the relief of symptoms associated with multiple sclerosis [20].

The commercialization of CBD in Romania adheres to current regulations concerning the safety and labeling of food supplements. In compliance with Law 339 of 2005, hemp-based products must not exceed a THC concentration of 0.2%. Key considerations for labeling and product testing include specifying the concentrations of CBD and THC, as well as providing clear instructions for use.

This discrepancy between marketing and scientific reality presents challenges for consumers and regulators alike. Many CBD products lack proper dosage standardization and quality control, leading to inconsistent results and unverified claims [17]. Consumers, drawn by aggressive marketing and anecdotal success stories, may be unaware of the scientific limitations surrounding CBD’s benefits. This article aims to critically examine the gap between the marketed claims of CBD supplements in Romania and the clinical reality. By exploring the available research and market trends, we highlight the urgent need for further investigation and stricter oversight to ensure that CBD products in Romania are safe, effective, and truly beneficial.

## 2. Materials and Methods

A comprehensive search for CBD-containing products available to Romanian consumers on all online pharmacy sites and most specialized online platforms was performed. Keywords such as “CBD” OR “cannabidiol” AND “dietary supplement” were used to select CBD products for this study. The products, and their concentration, indications and maximum recommended dose were registered. Only CBD-based supplements were selected, while cosmetic products were excluded. Supplements containing other cannabinoids or other active ingredients were also excluded. The search was performed in August 2024.

A systematic review of clinical trials evaluating the efficacy of CBD was conducted online using PubMed (https://pubmed.ncbi.nlm.nih.gov/) and the Cochrane Library. The systematic review was conducted in accordance with PRISMA guidelines. Only randomized placebo-controlled double-blind studies with a single treatment of CBD, e.g., studies with other cannabinoid add-on therapy or adjuvant regimes were excluded. Studies had to report dosage and dosage schemes to be included. The drugs used in clinical trials were either pharmaceutical CBD products or dosage forms compounded in accordance with GMP guidelines. Notably, in most clinical studies, formulations were specially prepared for patients to enable precise control over dosage and composition. Full-text articles were interdependently assessed by two reviewers. The following data were extracted: condition and symptoms, the year of publication, study population, the number of patients, the effect of treatment, dosage, administration form (pills, smoking, oil, etc.), and the length of treatment. Articles were limited to those published in English between 2000 and 2024 with no demographic limitation. However, other relevant studies, such as case studies or open-label trials providing valuable insights into the efficacy of CBD, have been taken into consideration.

The keywords and MeSH terms used were as follows: “cannabidiol” AND “clinical trial” OR “anxiety” OR “pain” OR “autism” OR “psychosis” OR “insomnia” OR “sleep” OR “cancer” OR “immun*”. The disorders included in our systematic review were chosen based on the most common indications listed on the labels of CBD supplements available in the Romanian market. The most relevant papers were selected after eligibility analysis and cross-checking.

## 3. Results

### 3.1. CBD-Based Products Available in Romania

**Figure 1:**
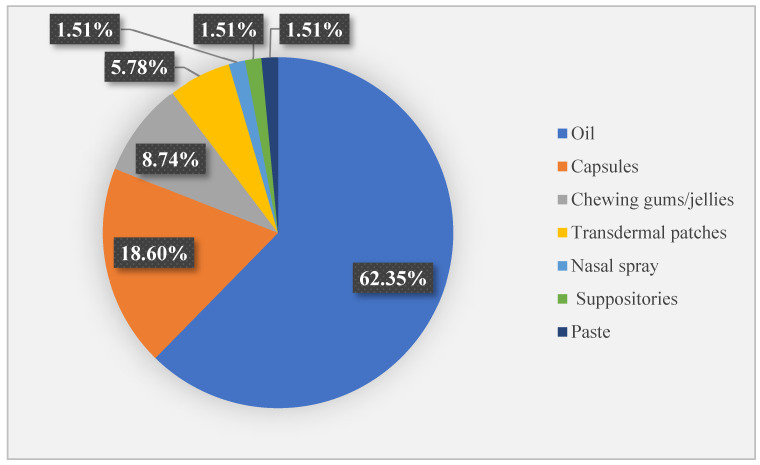
Pharmaceutical forms containing cannabidiol identified on the Romanian market.

In total, 76 CBD-containing supplements were identified (Figure 1), with most being oils with CBD concentrations between 5 and 40% (0.5–3 g/10 mL). Based on manufacturer dosing recommendations, these concentrations correspond to doses ranging from 2.5 mg/day to 70 mg/day. Only two products marketed as supplements had a higher concentration, 60%, with the recommended dose reaching 100–160 mg/day. For soft gel capsules filled with CBD oil, the recommended doses range from 5 to 75 mg/day. However, one product specifies only the total oil content (e.g., 498 mg oil per capsule) without indicating the exact CBD concentration, making it impossible to determine the actual CBD dosage.

Other forms and their corresponding doses include the following:

Suppositories: 50 mg per suppository per day.

Transdermal patches: 15 mg or 16 mg per patch per day.

Chewing gums: 8.33 mg or 10 mg per gum, with no specified maximum daily dose.

Jelly: 10 mg per day.

Paste: 50% concentration, with a recommended dose of 50 mg taken two to three times daily.

Nasal spray: Concentration not specified.

The products included in our study were selected based on the specific search criteria targeting online pharmacies and specialized websites accessible within the Romanian market. The selected products contain only CBD and excipients.

Our analysis focused on concentration percentages, as this information is commonly displayed by online retailers in Romania. CBD content labeling differs between Romanian supplements, which typically display concentration percentages, and clinical trial products, measured in mg per mL.

While some products are indicated by manufacturers for mood and overall wellness, many others include specific conditions in the labeling.

**Figure 2:**
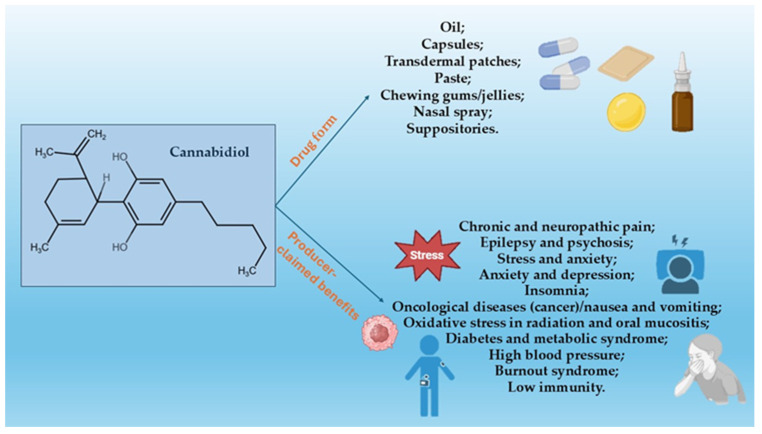
Producer-claimed benefits of cannabidiol supplements and drug forms available in Romania’s online market.

The indications specified by the manufacturers (Figure 2), when available, included the following:

- Chronic and neuropathic pain;
- Epilepsy and psychosis;
- Stress and anxiety (including post-traumatic stress);
- Anxiety and depression;
- Insomnia;
- Oncological diseases (cancer)/nausea and vomiting during chemotherapy;
- Oxidative stress in radiation and oral mucositis;
- Diabetes and metabolic syndrome;
- High blood pressure;
- Burnout syndrome;
- Neuropathy;
- Low immunity.

The maximum dose/day based on the recommendations of the manufacturers ranges between 2.5 and 160 mg/day, depending on the specific product. Dosing instructions and in some cases the benefits claimed by the producer for different conditions are listed on product labels. Most products formulated as oils, capsules, transdermal patches, and pastes are recommended for a range of health benefits, as previously outlined. Additionally, we identified four supplements that lack specific indications, comprising two capsule formulations and two chewing gum products. Furthermore, CBD nasal spray is marketed for its potential to enhance sleep quality, facilitate respiratory function, and promote relaxation, while suppositories are indicated for the restoration of hormonal balance.

### 3.2. Anxiety and Related Behavior

CBD has gained significant attention for its potential therapeutic effects in the treatment of anxiety disorders. Preclinical and clinical studies suggest that CBD modulates key molecular pathways involved in anxiety regulation [21]. One primary mechanism is through its interaction with the endocannabinoid system, particularly by enhancing the signaling of anandamide, an endogenous cannabinoid, which contributes to mood regulation and stress resilience [22]. Additionally, CBD acts as an agonist at serotonin 5-HT_1A_ receptors, a crucial pathway involved in anxiety and mood disorders [23]. CBD also exerts influence on the hypothalamic–pituitary–adrenal axis, reducing the release of stress hormones such as cortisol [21]. Given these effects, CBD supplements have become highly popular as an over-the-counter option for managing anxiety, with widespread commercial availability.

Case series [24] and open-label trials [25] report that dosages ranging from 25 mg/day to 800 mg/day reduce anxiety after a minimum of one month of therapy.

However, clinical trials do not entirely support these results. Studies investigating the effects of CBD on anxiety have produced varying results depending on the dosage and population. In healthy individuals, doses of 100 mg or 150 mg of oral CBD generally showed no significant reduction in anxiety compared to placebo, with no effects on blood pressure or cognitive function [26,27]. At higher doses of 300 mg, CBD is generally reported to decrease anxiety in both healthy individuals exposed to stress-inducing conditions and patients with anxiety disorders [28,29,30,31]. These studies, conducted in both simulated and real-world public speaking scenarios, demonstrated significant anxiolytic effects without impacting heart rate or blood pressure. However, the clinical randomized study by Stanley [27] reported CBD 300 and 600 mg did not significantly reduce anxiety in test anxiety in healthy volunteers [27]. In patients with social anxiety disorder and avoidant personality disorder, a 300 mg/day dose of CBD over four weeks produced anxiety reductions comparable to standard treatments like paroxetine. Doses of CBD over 10 mg/kg effectively reduced anxiety in other pathologies such as Parkinson’s disease [32] or autism spectrum disease [33].

In patients with post-traumatic stress disorder, the anxiolytic effect of CBD seems to depend on the nature of the trauma when patients recall their trigger event. Thus, Bolsoni et al. found that 300 mg of oral CBD did not reduce anxiety, alertness, discomfort, or blood pressure increases during traumatic recall in PTSD patients, but it significantly reduced cognitive impairment, with effects lasting for a week [34]. In a follow-up study, they reported that CBD effectively reduced anxiety and cognitive impairment in PTSD patients, but only for those with nonsexual trauma [35].

The findings of various double-blind clinical trials assessing the efficacy of cannabidiol (CBD) in treating anxiety are summarized in Table 1, while the results on treating secondary anxiety are detailed in Table 2.

### 3.3. Autism Spectrum and Behavioral Disorders

Cannabidiol has emerged as a promising candidate in the therapeutic landscape for autism spectrum disorder (ASD). Research into the molecular mechanisms underlying CBD’s potential efficacy in ASD suggests several pathways through which it may exert therapeutic effects: the interaction with the endocannabinoid system [21], modulating neurotransmitter release and synaptic plasticity [37], and the modulation of serotonin receptors [38]. These are implicated in mood regulation and social behavior [39], key areas often affected in autism. Preclinical studies have indicated that CBD may also affect neuroinflammation and oxidative stress, processes that are thought to contribute to ASD pathology [40]. By potentially modulating these molecular targets, CBD was hypothesized to provide a novel approach to managing the core symptoms and comorbid conditions associated with autism, warranting further investigation into its clinical efficacy and safety profile.

Several case reports [41,42] and observational studies [43,44] have reported positive outcomes for the use of cannabidiol in managing symptoms associated with ASD. These reports often highlight the potential benefits of CBD in improving behavior, social interactions, and overall quality of life in affected individuals. However, it is crucial to note that these positive outcomes have frequently been associated with the use of high doses of CBD (over 300 mg) or of combinations of CBD with THC. THC’s effects on human behavior are well-documented [45], and these properties may contribute significantly to the observed improvements in individuals with ASD.

We found only three double-blind, placebo-controlled, randomized controlled trials investigating the effects of cannabidiol in ASD, and one focusing on pediatric patients with severe behavioral problems (Table 3). A single dose of cannabidiol has notable effects on the glutamate–GABA systems and brain connectivity. However, its impact is highly context-dependent and varies significantly between neurotypical and autistic individuals. Specifically, CBD increases GABA+ levels in the basal ganglia (BG) and dorsomedial prefrontal cortex (DMPFC) in neurotypical individuals. In contrast, it decreases GABA+ levels in these same regions in autistic adults, with a particularly pronounced effect in the DMPFC [46,47]. Doses up to 1000 mg/day induced positive effects on behavioral problems in children and adolescents with intellectual disability. However, lower doses (10 mg/kg) did not impact social responsiveness in pediatric patients with ASD.

### 3.4. Pain

Cannabidiol (CBD) has shown potential as a treatment for pain, acting through several molecular mechanisms, particularly its effects on transient receptor potential vanilloid 1 (TRPV1) and its indirect modulation of the cannabinoid receptors CB_1_ and CB_2_. One key mechanism is CBD’s ability to modulate TRPV1 signaling, which is involved in pain sensation. Studies suggest that CBD inhibits neuronal hypersensitivity by reducing TRPV1 phosphorylation and desensitizing the receptor through the inhibition of the adenylyl cyclase-cAMP pathway [49]. By indirectly influencing CB_1_ and CB_2_ receptors, CBD modulates both central and peripheral pain pathways. CB_2_ activation, in particular, has been linked to the release of endogenous opioids like beta-endorphins, which play a role in reducing nociceptive signaling [49]. Moreover, CBD has been found to increase levels of the endogenous cannabinoids anandamide and 2-arachidonoylglycerol, further activating the cannabinoid system to modulate pain and inflammation [50]. CBD activates PPARγ receptors, which play a crucial role in the modulation of inflammatory pain [6]. Thus, it seems to reduce the release of pro-inflammatory molecules and alter pain signaling pathways, offering potential relief from chronic inflammatory pain.

Some case reports suggest CBD can reduce pain in various pathologies, including complex regional pain syndrome [51], cervical spondyloarthritis [52], or chronic temporomandibular arthralgia [53].

Recent clinical studies on the efficacy of CBD for pain management present mixed results, which vary by dosage and the duration of treatment (Table 4). In acute pain scenarios, Alaia et al. (2024) [54] observed that high doses of CBD (75 mg/day for patients under 80 kg and 150 mg/day for patients over 80 kg) provided significant pain relief only on the first day post-arthroscopic rotator cuff repair, with no sustained benefits through repeated administrations over two weeks. This suggests a potential for acute, but not chronic, efficacy at higher doses. Conversely, for chronic pain conditions, studies consistently show that CBD, across a spectrum of low to high doses (600 mg/day) [54,55,56,57,58,59], fails to provide effective relief, with no significant pain reduction compared to placebo. These findings suggest that while CBD may offer brief relief in acute scenarios at higher doses, it does not effectively alleviate chronic pain, casting doubt on its utility as a long-term analgesic.

### 3.5. Sleep Disorders

CB_1_ and CB_2_ receptors are widely distributed in brain regions associated with sleep regulation, such as the hypothalamus and brainstem. Their modulation by CBD promotes the regulation of the sleep–wake cycle. Additionally, CBD increases adenosine levels by inhibiting its reuptake, which may contribute to sleep promotion, as adenosine accumulation in the brain is linked to the onset of sleep and the regulation of circadian rhythms. By activating 5-HT_1A_ receptors, CBD alleviates the hyperarousal that disrupts sleep. Collectively, these molecular pathways suggest that CBD could serve as a multifaceted agent in improving sleep quality.

A retrospective case series (n = 25) at a psychiatric clinic investigated CBD’s efficacy in improving sleep as a supplementary treatment alongside standard care. Sleep scores improved initially in 66.7% of the patients but showed variability over time [59]. An open-label clinical trial demonstrated that the administration of CBD (maximum 25 mg/kg/day) significantly reduces sleep disruption in children with drug-resistant epilepsy (n = 35) [60]. These findings suggest potential benefits of CBD for anxiety-related disorders, underscoring the need for controlled clinical trials to further explore its therapeutic properties. A total of 38% (n = 8) of participants with PTSD reported subjective improvement in the quality of their sleep after 8 weeks of CBD administration [61].

Data from double-blind clinical trials that evaluate the effects of CBD on sleep quality and related disturbances are compiled in Table 5.

### 3.6. Psychosis

CBD appears to exert antipsychotic effects through non-D_2_ receptor pathways, distinguishing it from traditional antipsychotics. CBD inhibits the enzyme fatty acid amide hydrolase (FAAH), which degrades anandamide, an endogenous cannabinoid with antipsychotic properties. Thus, it increases anandamide levels in the brain, which is associated with symptom improvement in psychosis. This modulation of the endocannabinoid system is crucial, as it enhances the protective signaling pathways that regulate dopamine transmission and synaptic plasticity, both of which are dysregulated in psychotic disorders like schizophrenia [65].

Another important mechanism is CBD’s effect on serotonin 5-HT_1A_ receptors and TRPV1 channels, which are involved in neurocognitive processes related to psychosis. CBD has been shown to normalize brain activity in regions such as the hippocampus and prefrontal cortex, areas critical for cognitive function and psychotic symptoms. Through these interactions, CBD modulates brain circuitry implicated in motivational salience and memory, which can alleviate symptoms of psychosis by restoring functional connectivity and reducing hyperactivation in key brain regions [66].

The exploration of CBD as a treatment for psychosis has gained momentum through various open-label studies and case reports, indicating its potential efficacy. Initial investigations, including a notable case study involving a 19-year-old female with schizophrenia, demonstrated that CBD, administered at doses up to 1500 mg/day, resulted in a significant reduction of psychotic symptoms as assessed by the Brief Psychiatric Rating Scale [67]. Subsequent case series further supported these findings, with patients experiencing varying degrees of symptom relief while reporting minimal side effects, underscoring CBD’s tolerability [68]. However, systematic reviews indicate insufficient data to conclusively establish CBD’s effectiveness across diverse patient populations [68]. Ongoing clinical trials aim to clarify this issue.

Several clinical placebo-controlled, randomized, double-blinded trials explored the effects of CBD on various symptoms of schizophrenia and other psychotic disorders (Table 6). Single doses of CBD (300–600 mg) did not improve cognitive functions [69] nor influence precursory thinking or psychotic symptoms [70]. In contrast, Leweke et al. [65] demonstrated that increasing the CBD dosage progressively from 200 mg/day to 800 mg/day over four weeks was as effective as amisulpride in reducing positive psychotic symptoms and improving cognitive functions such as processing speed, visual memory, visuomotor coordination, and sustained attention. Other studies supported the idea that higher dosages (600–1000 mg/day) improved psychotic symptoms [71,72]. However, no cognitive enhancements [71] or only marginal improvements were observed [72]. These findings suggest a potential dosage-dependent response to CBD in the treatment of psychotic disorders, particularly for positive symptoms, but indicate limited effects on cognitive symptoms at the dosages studied.

### 3.7. Substance Addiction

CBD exerts multiple molecular effects that may support its therapeutic use in drug addiction. The inhibition of the reuptake and breakdown of anandamide leads to an enhancement of endocannabinoid signaling, which has been shown to modulate the reward system in the brain [73]. Furthermore, CBD acts as an allosteric modulator of μ- and δ-opioid receptors, which are closely involved in opioid addiction, suggesting it may reduce the rewarding effects of opioid use [74]. Additionally, CBD suppresses the release of pro-inflammatory cytokines, such as interleukin-1β and tumor necrosis factor-α, in key areas of the brain associated with addiction, including the prefrontal cortex and hippocampus. This anti-inflammatory action helps reduce the neuroinflammation-induced reinstatement of drug-seeking behavior, particularly in models of methamphetamine addiction [75]. Moreover, CBD’s interaction with D_2_-like dopamine receptors in the hippocampus suggests a role in regulating emotional memory and reward, contributing to its potential in mitigating drug cravings [76].

At a dose of 200 mg per day, CBD showed mixed results across addiction studies (Table 7). In cannabis use disorder, Haney et al. (2016) [77] found no impact on the subjective effects of cannabis, and Freeman et al. (2020) [78] reported no reduction in cannabis use. Similarly, 200 mg was ineffective in reducing crack-cocaine craving [79], though a lower dose of 400 µg via inhalation reduced cigarette consumption in nicotine addiction [80]. Higher doses of 400 mg and 800 mg yielded more consistent benefits. Freeman et al. (2020) [78] and Lees et al. (2023) [81] reported that both doses reduced cannabis use and improved working memory in cannabis use disorder. In heroin use disorder, 400 mg reduced cue-induced craving and anxiety [82], while 800 mg reduced alcohol craving and improved sleep and mood in alcohol use disorder [83]. However, 800 mg did not alleviate anxiety or cortisol levels in cocaine use disorder [84].

### 3.8. Immunity

CBD acts as an immunosuppressant and anti-inflammatory agent. CBD’s interaction with CB_2_ is critical for inhibiting pro-inflammatory processes and promoting regulatory immune responses, such as inducing apoptosis in immune cells through CB_2_-mediated pathways [86]. It induces regulatory T cells, suppressing T cell proliferation and contributing to immune tolerance [87]. Additionally, it suppresses the production of key cytokines such as interleukin-2 and interferon-gamma, which are essential for T cell activation and inflammatory responses, via non-CB_1/2_ dependent pathways [88]. CBD reduced systemic inflammation in models of acute conditions, such as acute lung injury, by decreasing leukocyte activation and cytokine production [89]. These effects extend to chronic inflammatory conditions, where CBD inhibits the activity of various immune cells, including macrophages and neutrophils, further highlighting its potential as a therapeutic agent in immune-modulated diseases.

Thus, it was investigated if CBD possesses beneficial effects in autoimmune diseases, particularly in inflammatory bowel diseases. Although extensive preclinical studies have demonstrated the positive effects of CBD in mouse and rat models of intestinal inflammation, clinical evidence is just starting to appear. Two clinical trials have explored the potential efficacy of cannabidiol (CBD) in treating inflammatory bowel diseases. In a study by Naftali et al. (2017), 19 patients with Crohn’s disease were administered 20 mg of CBD per day *as* sublingual oil over eight weeks in a randomized, placebo-controlled trial [90]. Although there was a reduction in disease activity at the end of the study, no significant difference was observed compared to the placebo group. In another randomized, placebo-controlled trial [91], 60 patients with mild to moderate ulcerative colitis were treated with oral CBD capsules at doses ranging from 50 mg to 250 mg per day over a 10-week period. While the primary endpoint of the study was not achieved, CBD showed potential benefits for the symptomatic treatment of ulcerative colitis.

### 3.9. Cancer

Preclinical data indicate that CBD inhibits cancer cell proliferation, induces cancer cell apoptosis, suppresses tumor metastasis, and modulates immune responses. CBD exerts these effects through multiple pathways, such as the activation of the endocannabinoid system, the inhibition of reactive oxygen species production, and the modulation of the PI3K/AKT/mTOR and MAPK pathways. These molecular actions have garnered interest in CBD’s potential to alleviate cancer-related symptoms, particularly pain, nausea, and inflammation [92,93].

A retrospective analysis of 119 cancer patients showed that 92% experienced clinical benefits, such as reductions in circulating tumor cells and solid tumor size, when treated with synthetic cannabidiol. There were no observed side effects [94]. In an open-label study, 43% of patients experienced significant symptom reduction, with drowsiness being the most common adverse effect. This trial confirmed the feasibility of using CBD (median dose 300 mg) for cancer-related symptoms, including nausea [95].

One double-blind, placebo-controlled trial found that CBD had a beneficial impact on the overall symptom burden in advanced cancer patients. Although the study was focused on a broad range of symptoms, including nausea, the results indicated that escalating doses up to 600 mg/day provided symptom relief with tolerable side effects [96]. Another randomized, placebo-controlled trial reported that CBD did not significantly reduce the total symptom distress score in cancer patients receiving palliative care. No notable differences were observed between CBD and placebo in terms of symptom relief or opioid use [97].

Most clinical trials evaluating CBD in cancer assess its antiemetic and analgesic effects in combination with THC, making it difficult to isolate CBD’s individual contribution [98,99]. This prevalent use of THC and CBD combinations in trials complicates the understanding of CBD’s specific therapeutic value in cancer management. While CBD shows potential in relieving cancer-related symptoms, particularly in high doses (300–600 mg/day), the clinical evidence remains mixed, with some studies showing benefits and others finding no significant improvements over placebo. Further studies with larger sample sizes and a focus on CBD alone are necessary to clarify its role in managing cancer-related emesis.

## 4. Discussion

This analysis of the marketing claims for CBD versus the results from preclinical and clinical trials highlights significant discrepancies, particularly concerning dosage, efficacy, and targeted conditions. Manufacturers promote CBD for a wide range of conditions, including chronic pain, anxiety, insomnia, and cancer-related symptoms, with recommended daily doses ranging from 2.5 mg to 160 mg. These marketed claims often lack alignment with evidence from clinical trials, which frequently show that higher doses of CBD (300–600 mg) are necessary for therapeutic effects, especially for conditions like anxiety [28], psychosis [65], and addiction [77,82,85].

For anxiety, manufacturers often market CBD at lower doses (e.g., 3–160 mg/day), yet clinical evidence suggests that only doses between 300 and 600 mg/day are effective in reducing anxiety in disorders like social anxiety and PTSD [28,29,30,31]. However, evidence on the optimal dosing of CBD for anxiety is still evolving, and the potential efficacy of lower doses cannot be ruled out. While doses of 100–150 mg have shown minimal effects, particularly in healthy individuals, benefits at lower doses (e.g., 30 mg/day) have been observed when using full-spectrum CBD products. These products contain additional cannabinoids and compounds that work synergistically, potentially enhancing the therapeutic effects of CBD at lower doses [100]. This contrast raises concerns about the potential mismatch between product labeling and the actual therapeutic outcomes currently observed. Similarly, in sleep disorders, marketed CBD products often claim efficacy for insomnia at lower doses, while trials show that only high doses provide significant benefit, particularly for patients with disrupted sleep patterns [62,63,64].

In the treatment of chronic pain, manufacturers claim CBD’s effectiveness in conditions like neuropathy and inflammatory pain. However, clinical trials demonstrate mixed results. While higher doses (75–150 mg) provide short-term relief after surgery [54], CBD appears less effective for the long-term management of chronic pain [56,58,59]. This inconsistency raises doubts about the widespread marketing of CBD as a reliable analgesic for chronic conditions. For cancer-related symptoms, including nausea and pain, the trials often involve combinations of CBD with THC [98,99], complicating the ability to attribute therapeutic effects solely to CBD.

Some marketed uses, such as “low immunity” or “burnout syndrome,” lack robust scientific backing. For example, while CBD has demonstrated anti-inflammatory effects and immunomodulatory potential in preclinical studies, these findings do not yet translate into conclusive clinical evidence for immune-related conditions. The claim that CBD boosts “low immunity” is particularly unsupported, as trials on CBD’s effect on the immune system focus on its immunosuppressive properties rather than the enhancement of immunity [90,91]. This is evident in its proposed role in autoimmune diseases and chronic inflammatory conditions, where it suppresses inflammation, not boosts immune function.

For psychotic disorders, doses of 800 mg/day demonstrated similar efficacy to standard antipsychotics in reducing positive symptoms [66], yet marketed doses for general wellness are much lower and unlikely to offer such benefits.

A critical issue is the lack of standardization in CBD product labeling, particularly in capsules and oils. Manufacturers frequently list the total milligrams of oil or product without specifying the concentration of CBD per dose, leaving consumers to calculate the effective amount. This lack of transparency can mislead users regarding the therapeutic potential of CBD and its required dosing. Clinical trials show that precise dosing, especially for higher doses, is essential for achieving desired therapeutic effects in conditions like addiction, cancer-related symptoms, and chronic pain.

## 5. Conclusions

The disparity between marketing claims for CBD products and clinical trial evidence highlights deeper issues in the broader regulation of supplements. While prescription cannabinoids, when administered under appropriate medical supervision, may strike a balance between benefit and risk, the current landscape of CBD supplements complicates this. Dosing guidelines are frequently unclear, product contents vary, and the effects remain difficult to predict due to a lack of comprehensive data. This creates significant challenges for healthcare providers trying to oversee supplement use, particularly when patients often fail to report their use of these products. Furthermore, supplements and pharmaceuticals are held to vastly different standards, leaving patients to make uninformed choices in the face of limited or ambiguous data. This situation calls for stronger regulation, improved communication between patients and providers, and a commitment to ensuring that all therapeutic claims are grounded in reliable, consistent evidence.

## Figures and Tables

**Table 1:** Double-blind clinical trials assessing the efficacy of cannabidiol in anxiety.

Author	Patients	Study Design	Administered Dose	Pharmaceutical Form	Conclusions
Bergamaschi M.M. et al., 2011 [28]	Social anxiety disorder (n = 24)	Randomized, parallel-group, double-blind, placebo-controlled trial	600 mg; single dose	Oral gelatin capsules	CBD significantly reduced anxiety, cognitive impairment, discomfort, and alertness during simulation public speaking test.
Crippa J.A.S. et al., 2011 [28]	Social anxiety disorder patients (n = 10)	Randomized, double-blind, placebo-controlled trial	400 mg; single dose	Oral gelatin capsules	Acute administration reduced subjective anxiety.
Masataka N., 2019 [30]	Social anxiety disorder (n = 37)	Randomized, double-blind, placebo-controlled trial	300 mg/day; 4 weeks	CBD oil 21.4/mL	CBD significantly decreased anxiety measured by both Fear of Negative Evaluation Questionnaire and Liebowitz Social Anxiety Scale.
Gournay L.R, et al., 2021 [31]	Individuals with elevated trait worry (n = 63)	Randomized, double-blind, placebo-controlled study design	300 mg/day; 50 mg/day acute/2 weeks	Soft gel capsules	CBD 50/300 mg did not effectively reduce worry severity in the context of acute or repeated administration. However, repeated 300 mg CBD administration for 2 weeks, but not an acute 300 mg dose, reduced anxiety symptoms compared to placebo.

**Table 2:** Double-blind clinical trials assessing the efficacy of cannabidiol in secondary anxiety.

Author	Patients	Study Design	Administered Dose	Pharmaceutical Form	Conclusions
de Faria S.M. et al., 2020 [32]	Patients with Parkinson’s disease undergoing a simulated public speaking test (n = 24)	Randomized, double-blind, placebo-controlled, crossover clinical trial	300 mg/day; single dose	Soft gel capsules	Acute CBD administration at a dose of 300 mg decreased anxiety in patients with PD, and there was also decreased tremor amplitude in an anxiogenic situation.
Parrella N-F et al., 2024 [33]	Autism spectrum disorder (children; n = 29)	Randomized double-blind, placebo-controlled, crossover clinical trial	10 mg/kg/day; 12 weeks	CBD oil 100 mg/mL	Reduces anxiety symptoms assessed using PROMIS Anxiety and DBC-2 Anxiety scales.
Bolsoni L.M. et al., 2022 [34]	Post-traumatic stress disorder (n = 33)	Randomized, parallel-group, double-blind, placebo-controlled trial	300 mg CBD; single dose	Oral capsules	No significant impact on anxiety levels, alertness, or discomfort during trauma recall. CBD did not significantly alter physiological markers of anxiety, such as blood pressure, heart rate, and salivary cortisol levels. CBD significantly reduced cognitive impairments (such as confusion or difficulty in thinking) during the recall of traumatic events.
Telch M.J., 2022 [36]	Patients with post-traumatic stress disorder (n = 150)	Phase II randomized double-blind placebo-controlled fixed dose clinical trial.	300 mg synthetic CBD	Oil formulations	PTSD symptom severity, patient-rated depression, overall disability, anxiety, quality of life, and alcohol use—results not reported.

**Table 3:** Double-blind clinical trials assessing the efficacy of cannabidiol in autism spectrum and behavioral disorders.

Author	Patients	Study Design	Administered Dose	Pharmaceutical Form	Conclusions
Pretzsch C.M. et al., 2019a [47]	Autism spectrum disorder (adults; n = 34)	Randomized placebo-controlled single dose trial	600 mg CBD; single dose	“liquid oral dose” (pharmaceutical form not specified)	CBD modulates glutamate–GABA systems, but prefrontal GABA systems respond differently in autism spectrum disorder. In contrast, CBD increased GABA+ levels in the BG and DMPFC voxel in neurotypicals, but decreased GABA+ levels in the BG and (markedly so) in the DMPFC voxel of autistic adults.
Pretzsch C.M. et al., 2019c [46]	Autism spectrum disorder (adults; n = 34)	Randomized placebo-controlled single dose trial	600 mg CBD; single dose	“liquid oral dose” (pharmaceutical form not specified)	Especially in ASD, CBD alters regional fALFF and FC in/between regions consistently implicated in ASD. fALFF ‘fractional amplitude of low-frequency fluctuations; FC, functional connectivity.
Efron D. et al., 2021 [48]	Severe behavioral problems in children and adolescents with intellectual disability (n = 8)	Double-blind, placebo-controlled, two-armed, parallel-design, randomized trial	Up-titration over 9 days to 20 mg/kg/day; maximum dose 1000 mg/day	98% CBD 100 mg/mL in grapeseed oil or placebo orally	Significant reduction in irritability, social withdrawal, stereotypic behavior, hyperactivity/non-compliance, and inappropriate speech.
Parrella N.-F. et al., 2024 [33]	Autism spectrum disorder (children; n = 29)	Double-blind, placebo-controlled randomized, crossover design	CBD 10 mg/kg/day; 12 weeks	CBD oil 100 mg/mL	No significant effect observed for the Social Responsiveness Scale-2. Significant improvements were observed in secondary measures of social functioning (PROMIS-Social and DBC-2 Social Relating). Reduction of parental stress.

**Table 4:** Double-blind clinical trials assessing the efficacy of cannabidiol in non-cancer pain.

Author	Patients	Study Design	Administered Dose	Pharmaceutical Form	Conclusions
Notcutt et al., 2004 [55]	Various forms of chronic non-cancer pain (n = 34)	Double-blind, placebo-controlled, randomized crossover trial	2.5 to 15 mg/day; 12 weeks	Sublingual spray (synthetic CBD)	CBD did not reduce pain compared to placebo.
Vela et al., 2021 [56]	Patients with hand osteoarthritis and psoriatic arthritis (n = 136)	Randomized, double-blind, placebo-controlled design	20 to 30 mg; 12 weeks	Capsules (synthetic CBD)	No change vs. placebo in pain intensity during the past 24 h using various scales
Bebee et al., 2021 [57]	Lower back pain (n = 100)	Randomized, double-blind, placebo-controlled, between-subjects design	400 mg; single dose	Capsules (synthetic CBD)	Verbal numerical pain scale (range, 0–10)
Pramhas et al., 2023 [58]	Painful chronic osteoarthritis of the knee (n = 86)	Prospective, randomized, placebo-controlled, double-blind, parallel-group study.	600 mg/day; 8 weeks	Capsules (synthetic CBD)	In patients with knee osteoarthritis, oral high-dose add-on cannabidiol had no additional analgesic effect compared to adding placebo to continued paracetamol. These results do not support the use of cannabidiol as an analgesic supplement for knee osteoarthritis.
Zubcevic K. et al., 2023 [59]	Painful polyneuropathy, post-herpetic neuralgia and peripheral nerve injury failing at least one previous evidence-based pharmacological treatment (n = 115)	Placebo-controlled, randomized, double-blind trial	5–50 mg/day; 8 weeks	Capsules (synthetic CBD)	Did not relieve peripheral neuropathic pain in patients failing at least one previous evidence-based treatment for neuropathic pain.
Alaia M.J. et al., 2024 [54]	Postoperative pain control after arthroscopic rotator cuff repair (n = 83)	Multicenter, placebo-controlled, randomized, double-blind trial	75 mg/day for patients < 80 kg; 150 mg/day for patients > 80 kg; 14 days	Capsules (synthetic CBD)	A significant difference in VAS pain score vs. placebo was only observed on day 1. No significant difference vs. placebo was observed after repeated administrations.

**Table 5:** Double-blind clinical trials assessing the efficacy of cannabidiol in sleep disorders.

Author	Patients	Study Design	Administered Dose/Dosage Form	Conclusions
Saleska J.L. et al., 2023 [62]	Sleep disturbances (n = 1793)	Randomized, double-blind controlled trial	15 mg CBD; 4 weeks; oral capsules	CBD significantly reduced sleep issues, with no added benefits from cannabinol or cannabichromene. Formulations with melatonin and CBD improved some sleep aspects, but overall effectiveness was similar to CBD alone.
Wang M., 2024 [63]	Primary insomnia (n = 125)	Double-blind, placebo-controlled, randomized crossover clinical trial	CBD (300 mg) and terpenes; ≥4 days/week over 4 weeks; oral capsules	CBD increased the mean nightly percentage of time spent in slow-wave sleep plus rapid eye movement sleep compared to placebo, particularly in participants with low baseline slow-wave sleep plus rapid eye movement sleep and day sleepers, without affecting total sleep time, heart rate, or heart rate variability, or causing adverse events.
Narayan A., 2024 [64]	Primary insomnia (n = 30)	Randomized, placebo-controlled, parallel design	150 mg CBD/day; 3 weeks; CBD oral solution 100 mg/mL	Insomnia severity, sleep-onset latency, sleep efficiency, and wake after sleep onset were not influenced by CBD treatment. CBD improved well-being, transiently elevated behavior following wakefulness, and had superior objective sleep efficiency compared to placebo.

**Table 6:** Double-blind clinical trials assessing the efficacy of cannabidiol in sleep disorders.

Author	Patients	Study Design	Administered Dose	Pharmaceutical Form	Conclusions
Hallak J.E. et al., 2010 [69]	Schizophrenia (n = 28)	Placebo-controlled, randomized, double-blind parallel trial	300 or 600 mg; single dose	Oral capsules	No cognitive improvement was observed.
Leweke F.M. et al., 2012 [65]	Acute psychosis (n = 32)	Placebo-controlled, randomized, double-blind parallel trial	200 mg/day up to 800 mg/day; 4 weeks	Oral capsules	CBD is as effective as amisulpride in improving psychotic symptoms, processing speed, visual memory, visuomotor coordination, and sustained attention.
Boggs D.L. et al., 2018 [71]	Chronic schizophrenia (n = 42)	Placebo-controlled, randomized, double-blind parallel trial	600 mg/day; 6 weeks	Oral capsules	Psychotic symptoms improved in both groups (CBD and placebo) without significant difference.
McGuire P. et al., 2018 [72]	Schizophrenia (n = 88)	Placebo-controlled, randomized, double-blind parallel trial	1000 mg/day; 6 weeks	Oral solution	Treatment with CBD improved positive psychotic symptoms and slightly improved cognitive performance vs. placebo, although the latter did not reach statistical significance.
Hundal H. et al., 2018 [72]	Paranoia (n = 32)	Placebo-controlled, randomized, double-blind parallel trial	600 mg; single dose	Oral capsules	CBD did not impact precursory thinking and psychotic symptoms.

**Table 7:** Double-blind clinical trials assessing the efficacy of cannabidiol in substance addiction.

Author	Patients	Study Design	Administered Dose	Pharmaceutical Form	Conclusions
Cannabis use disorder					
Haney M. et al., 2016 [77]	Healthy cannabis smokers (n = 32)	Placebo-controlled, randomized, double-blind cross-over trial	200, 400, and 800 mg; single dose	Oral capsules	CBD did not alter the subjective effects of smoked cannabis.
Freeman T.P. et al., 2020 [78]	Cannabis use disorder (n = 59)	Randomized, double-blind, placebo-controlled, parallel design	200 mg/day; 4 weeks	Oral capsules	Doses of 400 mg and 800 mg, but not 200 mg, of CBD were effective in reducing cannabis use.
Lees R. et al., 2023 [81]	Moderate or severe DSM-5 cannabis use disorder (n = 70)	Phase 2a randomized, double-blind, placebo-controlled, parallel clinical trial	200 mg, 400 mg, and 800 mg; 4 weeks	Oral capsules	CBD did not influence delayed verbal memory. CBD did not have broad cognitive effects, but 800 mg daily treatment improved working memory manipulation.
Nicotine addiction					
Morgan et al., 2013 [80]	Tobacco smokers (n = 24)	Randomized, double-blind, placebo-controlled, parallel trial	400 µg; 1 week	Inhalation/vaporized	CBD reduced the number of cigarettes smoked during treatment and at follow-up.
Hindocha et al., 2018 [85]	Tobacco smokers (n = 30)	Randomized, double-blind, placebo-controlled, parallel trial	800 mg; single dose	Oral capsules	CBD reduced the salience and pleasantness of cigarette cues, compared with placebo, but did not influence tobacco craving or withdrawal.
Other addictions					
Hurd Y.L. et al., 2019 [82]	Heroin use disorder (n = 14)	Randomized, double-blind, placebo-controlled, cross-over design	400 mg/day; 3 days	CBD oral solution (100 mg/mL; Epidiolex)	CBD reduces cue-induced craving and anxiety.
de Meneses-Gaya et al., 2020 [79]	Crack-cocaine dependence (n = 31)	Randomized, double-blind, placebo-controlled, parallel design	300 mg/day; 10 days	Oral capsules	CBD did not reduce craving vs. placebo. It did not improve anxiety, depression, and sleep.
Mongeau-Pérusse V. et al., 2022 [84]	Individuals with cocaine use disorder (n = 78)	Randomized, double-blind, placebo-controlled trial	Max. 800 mg/day; 12 weeks		CBD 800 mg did not reduce anxiety symptoms and cortisol levels in individuals with cocaine use disorder.
Hurzeler et al., 2024 [83]	Non-treatment seekers with alcohol use disorders	Randomized, double-blind, placebo-controlled, cross-over study	800 mg/day; 3 days	Gel capsules	CBD reduced alcohol craving and seeking. It improved clinical characteristics leading to relapse such as sleep and mood disturbances.

## Data Availability

All data generated or analyzed during this study are included in this published article.
